# Master Regulator for Chondrogenesis, Sox9, Regulates Transcriptional Activation of the Endoplasmic Reticulum Stress Transducer BBF2H7/CREB3L2 in Chondrocytes*

**DOI:** 10.1074/jbc.M113.543322

**Published:** 2014-04-06

**Authors:** Kenta Hino, Atsushi Saito, Miori Kido, Soshi Kanemoto, Rie Asada, Tomoko Takai, Min Cui, Xiang Cui, Kazunori Imaizumi

**Affiliations:** From the Department of Biochemistry, Institute of Biomedical & Health Sciences, University of Hiroshima, 1-2-3 Kasumi, Minami-ku, Hiroshima 734-8553, Japan

**Keywords:** Cartilage, Cell Biology, Chondrogenesis, Protein Secretion, Transcription Factors, BBF2H7, ER Stress Transducer, Sox9

## Abstract

The endoplasmic reticulum (ER) stress transducer, box B-binding factor 2 human homolog on chromosome 7 (BBF2H7), is a basic leucine zipper (bZIP) transmembrane transcription factor. This molecule is activated in response to ER stress during chondrogenesis. The activated BBF2H7 accelerates cartilage matrix protein secretion through the up-regulation of Sec23a, which is responsible for protein transport from the ER to the Golgi apparatus and is a target of BBF2H7. In the present study, we elucidated the mechanisms of the transcriptional activation of *Bbf2h7* in chondrocytes. The transcription of *Bbf2h7* is regulated by Sex determining region Y-related high-mobility group box 9 (Sox9), a critical factor for chondrocyte differentiation that facilitates the expression of one of the major cartilage matrix proteins Type II collagen (Col2), through binding to the Sox DNA-binding motif in the *Bbf2h7* promoter. BBF2H7 is activated as a transcription factor in response to physiological ER stress caused by abundant synthesis of cartilage matrix proteins, and consequently regulates the secretion of cartilage matrix proteins. Taken together, our findings demonstrate novel regulatory mechanisms of Sox9 for controlling the secretion of cartilage matrix proteins through the activation of BBF2H7-Sec23a signaling during chondrogenesis.

## Teaser

**Background:** BBF2H7 promotes cartilage matrix protein secretion through activation of Sec23a expression during chondrogenesis.

**Results:** Sox9 induces BBF2H7 expression, followed by accelerating secretion of cartilage matrix proteins in chondrocytes.

**Conclusion:** Sox9 simultaneously induces *Bbf2h7* and *Col2*, followed by promoting secretion of Col2 through activation of BBF2H7-Sec23a signaling.

**Significance:** This might be the first to define mechanisms for cartilage matrix protein secretion regulated by Sox9.

## Introduction

Eukaryotic cells have a specific signal transduction system to adapt to the endoplasmic reticulum (ER)^3^ dysfunction caused by accumulation of unfolded and/or misfolded proteins in the ER lumen. This system is collectively known as the unfolded protein response (UPR) (1, 2). Recent studies have shown that the UPR signaling is involved not only in adapting to accumulation of unfolded proteins but also in regulation of physiological functions and homeostasis including cell differentiation and tissue formation (3–5). In mammalian cells, the three major transducers of UPR are double-strand RNA-dependent protein kinase (PKR)-like ER kinase (PERK) (6), inositol-requiring kinase 1 (IRE1) (7, 8), and activating transcription factor 6 (ATF6) (9). Furthermore, novel types of ER stress transducers that share a region of high sequence similarity with ATF6 have been identified (10, 11). These new members include OASIS/CREB3L1 (12, 13), BBF2H7/CREB3L2 (14, 15), CREBH/CREB3L3 (4, 16), CREB4/CREB3L4 (17, 18), and Luman/CREB3 (19).

*Bbf2h7* was originally identified by its involvement in gene translocation in low-grade fibromyxoid sarcoma as a result of a fusion event between the *fused in sarcoma* gene on chromosome 16 and the *Bbf2h7* gene on chromosome 7 (20). BBF2H7 is preferentially expressed in chondrocytes of developing cartilage, and activated in response to ER stress and differentiating stimuli (15). This molecule is localized at the ER membrane under normal conditions, and is easily degraded via the ubiquitin-proteasome pathway (21). Under ER stress conditions, BBF2H7 stability is enhanced. The stabilized BBF2H7 is automatically transported from the ER to the Golgi apparatus and then cleaved at the site-1-protease (S1P) site by S1P and subsequently at the transmembrane domain containing the site-2-protease (S2P) site by S2P (21–23). The processed N-terminal fragments translocate into the nucleus to promote transcription of target genes (14, 15). *Bbf2h7*-deficient (*Bbf2h7*^−/−^) mice exhibit severe chondrodysplasia and die from suffocation shortly after birth, because of an immature chest cavity (15). Proliferating chondrocytes of *Bbf2h7*^−/−^ mice show abnormal expansion of the ER that contains aggregated cartilage matrix proteins. We have demonstrated that in chondrocytes, BBF2H7 is activated in response to physiological ER stress caused by production of abundant cartilage matrix proteins during chondrogenesis (15). The cleaved BBF2H7 N terminus acts as a transcription factor to activate the expression of Sec23a, which is responsible for protein transport from the ER to the Golgi apparatus (15). The accumulation of cartilage matrix proteins in the ER lumen of *Bbf2h7*^−/−^ proliferating chondrocytes is caused by the reduction in Sec23a expression. These previous findings indicate that BBF2H7 contributes to the substantial growth of developing cartilage through the acceleration of cartilage matrix protein secretion during chondrogenesis. However, the transcriptional regulation of *Bbf2h7* is still unknown.

Sex determining region Y (SRY)-related high-mobility group (HMG) box 9 (Sox9) belongs to the HMG-box DNA-binding proteins sharing a high degree of homology with the mammalian testis-determining factor, SRY. Sox proteins including Sox9 have critical functions in a number of developmental processes, such as sex determination, skeleton formation, pre-B and T cell development and neural induction, that facilitate the expression of target genes through binding to the Sox DNA-binding motif ([A/T][A/T]CAA[A/T]) as a transcription factor (24–26). Sox9 is expressed in all chondroprogenitors and differentiated chondrocytes, but not in hypertrophic chondrocytes (27, 28), and is essential for differentiating chondrogenic mesenchymal condensations into chondrocytes, as well as regulating at every stage of chondrocyte differentiation (29–31). Previous studies based on mesenchymal cells of the *Sox9*-deficient (*Sox9*^−/−^) lineage showed that *Sox9*^−/−^ embryonic stem cells were excluded from chondrogenic mesenchymal condensations and could not express cartilage matrix genes including *Type II collagen* α*1* (*Col2*α*1*), *Type XI collagen* α*2* (*Col11*α*2*), and *Aggrecan* (*Agn*) (29). Other studies have revealed that Sox9 bound to chondrocyte-specific enhancer elements in *Col2*α*1*, *Col11*α*2*, and *Agn*, to activate their expression (32–35), indicating that they are direct targets of Sox9. Selective deletion of *Sox9* from chondrocytes at later stages of development, the resultant chondrocytes exhibited decreased proliferation and inhibited expression of cartilage matrix genes and downstream genes of Indian hedgehog (Ihh), including parathyroid hormone-related protein (PTHrP) (30). Furthermore, proliferating chondrocytes seem to prematurely convert to hypertrophic chondrocytes. Thus, Sox9 is a critical factor for all phases of the chondrocyte lineage from early condensations to the differentiation of proliferating chondrocytes into hypertrophic chondrocytes.

Here, we found that the promoter region of *Bbf2h7* contains the Sox DNA-binding motif. Sox9 promotes the expression of *Bbf2h7* through binding to this element in chondrocytes, followed by promoting the secretion of cartilage matrix proteins. Our findings revealed novel molecular mechanisms of cartilage matrix protein secretion regulated by the Sox9-BBF2H7-Sec23a axis.

## EXPERIMENTAL PROCEDURES

### 

#### 

##### Mice

C57BL/6 mice and *Bbf2h7*^−/−^ mice (established in our laboratory) (15) were used in this study. The experimental procedures and animal housing conditions were approved by the Committee of Animal Experimentation, Hiroshima University.

##### Cell Culture, Transfection, and Adenoviruses

Primary cultured chondrocytes were prepared from rib cartilage of embryonic day (E) 18.5 wild-type (WT) and *Bbf2h7*^−/−^ littermate mice using previously published protocols (15). Briefly, chondrocytes were isolated using 0.2% collagenase D (Roche) after adherent connective tissue was removed by 0.2% trypsin (Sigma) and collagenase D pretreatment. Isolated chondrocytes were maintained in α-MEM (Invitrogen) supplemented with 10% FCS and 50 μg/ml ascorbic acid. Micromass culture was performed previously published protocols (15). Briefly, mesenchymal cells were prepared from the limbs of E11.5 mice and digested with 0.1% trypsin and 0.1% Collagenase D. A total of 1 × 10^7^ cell/ml were plated and maintained in α-MEM supplemented with 100 ng/ml BMP-2 (Sigma), 50 μg/ml ascorbic acid and 5 nm β-glycerophosphate. ATDC5, a murine chondrogenic cell line, was obtained from RIKEN Cell Bank (Ibaraki, Japan) and cultured in α-MEM supplemented with 10% FCS and ITS solution (Sigma). Primary cultured NPCs were prepared from E14.5 mouse telencephalon using previously published protocols (36). Briefly, the telencephalons of E14.5 mice were triturated in Hank's balanced salt solution (Invitrogen) by mild pipetting with a 1-ml pipette tip (Gilson, Greiner Bio-one). Dissociated cells were cultured in 1.27 g/liter NaHCO_3_, 25 mg/liter insulin (Sigma), 100 mg/liter apo-transferrin (Sigma), 16 mg/liter putrescin (Sigma), 30 nm sodium selenite (Sigma), 20 nm progesterone (Sigma) in DMEM-F12 (Invitrogen). For knockdown of *Sox9*, primary cultured chondrocytes were transfected with a *Sox9*-targeting siRNA (Thermo Scientific; d-059108–02-0002) or a non-targeting siRNA (Thermo Scientific; d-001206–13-05) as a control using Lipofectamine 2000 reagent (Invitrogen). The recombinant adenovirus-carrying mouse HA-Sox9 and BBF2H7 N terminus were used previously (15). The recombinant adenoviruses-carrying mouse Sox5 and Sox6 were kindly gifted by Dr. Riko Nishimura (Osaka University).

##### In Situ Hybridization

*In situ* hybridization was performed using digoxigenin-labeled *Bbf2h7* and *Sox9* cRNA probes (15). Antisense probes were prepared by *in vitro* transcription in the presence of digoxigenin-labeled dUTP using various cDNAs subcloned into pGEM-Teasy vectors (Promega) as templates. Epiphyseal regions of tibiae at E18.5 mice were frozen immediately and sectioned (5 μm). Serial frozen sections were fixed for 20 min with 4% formalin and then treated with 0.1% proteinase K for 10 min. After washing with PBS, the sections were re-fixed for 20 min with 4% formalin in PBS and then treated with 0.1 m triethanolamine and 2.5% anhydrous acetic acid in diethylpyrocarbonate (DEPC)-treated water for 10 min followed by washing with PBS. The sections were prehybridized for 1 h at 37 °C in hybridization buffer (0.01% dextran sulfate, 0.01 m Tris-HCl, pH 8.0, 0.05 m NaCl, 50% formamide, 0.2% sarcosyl, 1× Denhardt's solution, and 0.2 mg/ml salmon testis DNA), and then hybridized overnight at 55 °C in hybridization solution with 100 ng/ml cRNA probe. After washing with 4×saline sodium citrate (SSC) buffer (1×SSC: 0.15 m NaCl and 0.015 m sodium citrate, pH 7.0) for 20 min at 60 °C, the sections were washed with 2× SSC buffer and 50% formamide in DEPC-treated water for 30 min at 60 °C. Sections were treated with RNase A in RNase buffer (10 mm Tris-HCl, pH 7.4, 1 mm EDTA, pH 8.0, and 0.5 m NaCl) for 15 min at 37 °C to remove unhybridized probes. After RNase treatment, the sections were washed with 2×SSC buffer and 50% formamide in DEPC-treated water for 30 min at 60 °C, and then treated with 1.5% blocking reagent (Roche) in 100 mm Tris-HCl, pH 7.5, and 150 mm NaCl for 1 h at room temperature. To detect digoxigenin-labeled cRNA probes, an anti-digoxigenin antibody conjugated with alkaline phosphatase (Roche) was used at a dilution of 1:500, and then the color was developed by incubation in a solution of 4-nitro blue tetrazolium chloride (Wako) and 5-bromo-4-chloro-3-indolyl phosphate (Roche).

##### Western Blotting

Proteins were extracted from primary cultured chondrocytes and primary cultured NPCs using a cell extraction buffer containing 0.9% SDS, 15 mm EDTA, pH 8.0, 8 mm methionine and a protease inhibitor mixture (Calbiochem). The lysates were incubated on ice for 45 min. After centrifugation at 16,000 × *g* for 15 min, the protein concentrations of the supernatants were determined by a BCA assay kit (Thermo Scientific). Equal amounts of proteins were subjected to SDS-PAGE. For immunoblotting, the following antibodies and dilutions were used: anti-β-actin (1:3,000; Sigma), anti-BBF2H7 (1:1,000) (15), anti-Sox9 (1:250; Santa Cruz Biotechnology), anti-Sox5 (1:1,000; Abcam), anti-Sox6 (1:1,000; Abcam), anti-GFAP (1:1,000; Sigma), anti-S100β (1:1,000; Abcam), anti-Ihh (1:1,000; Abcam), anti-Col2 (1:500; Acris Antibodies GmbH), anti-Col11 (1:500; Origene), anti-Sec23a (1:1,000; Sigma), and anti-Col10 (1:1,000; Calbiochem). The density of each band was quantified using Adobe Photoshop Elements 2.0.

##### Immunofluorescence

Primary cultured chondrocytes were fixed in cold MeOH and then permeabilized in 0.5% Triton X-100. The following antibodies and dilutions were used: anti-Sox9 (1:250; Santa Cruz Biotechnology) and anti-BBF2H7 (1:500) (15). Cells were visualized under a confocal microscope (FV1000D, Olympus).

##### Luciferase Assay

ATDC5 cells were plated and transfected with 0.2 μg of a pGL3 basic reporter plasmid carrying the firefly luciferase gene (Promega) and the referenced constructs and pRL-SV40, carrying the *Renilla* luciferase gene under the control of the SV40 enhancer and promoter (Promega), using Lipofectamine 2000 reagent. After 24 h, luciferase activities were measured with Dual-Luciferase Reporter Assay System (Promega) and GloMax Multi+ Detection System (Promega), according to the manufacturer's protocol. Relative activity was defined as the ratio between firefly luciferase activity and that of *Renilla* luciferase.

##### Electrophoresis Mobility Shift Assay

The electrophoretic mobility shift assay was performed using Chemiluminescent Nucleic Acid Detection Module (Thermo Scientific), according to the manufacturer's protocol. The sequences of the oligonucleotides used in the binding were: 5′-GGTAACCTTGTCTCAAAAACAAAACAACAAAATCCTATGA-3′ (Sox-wt) and 5′-GGTAACCTTGTCTCAAAAACgcAACAACAAAATCCTATGA-3′ (Sox-mt).

##### Chromatin Immunoprecipitation Assay

The chromatin immunoprecipitation assay was performed as previously described (12). The primers used for the mouse *Bbf2h7* promoter were: 5′-GAGGGATCAGGAGTTCAAGCTCAG-3′ (forward) and 5′-GACATTAGCCAATCTTGCCACAC-3′ (reverse), yielding a 176-bp product. The primers used for the mouse *Col2*α*1* enhancer were: 5′-CAGCGATGGCTTCCAGATGGGCTG-3′ (forward) and 5′-GAGGTGGCGGCAGGCGGGCAC-3′ (reverse), yielding a 222-bp product. The following antibodies were used: anti-Sox9 (Santa Cruz Biotechnology), anti-histone H3 (Santa Cruz Biotechnology), and rabbit IgG (Sigma).

##### Statistical Analysis

Statistical comparisons were made using the unpaired Student's *t* test. *p* values of less than 0.05 were considered statistically significant.

## RESULTS

### 

#### 

##### BBF2H7 and Sox9 Are Expressed in Resting Chondrocytes and Proliferating Chondrocytes during Chondrogenesis

**FIGURE 1.**
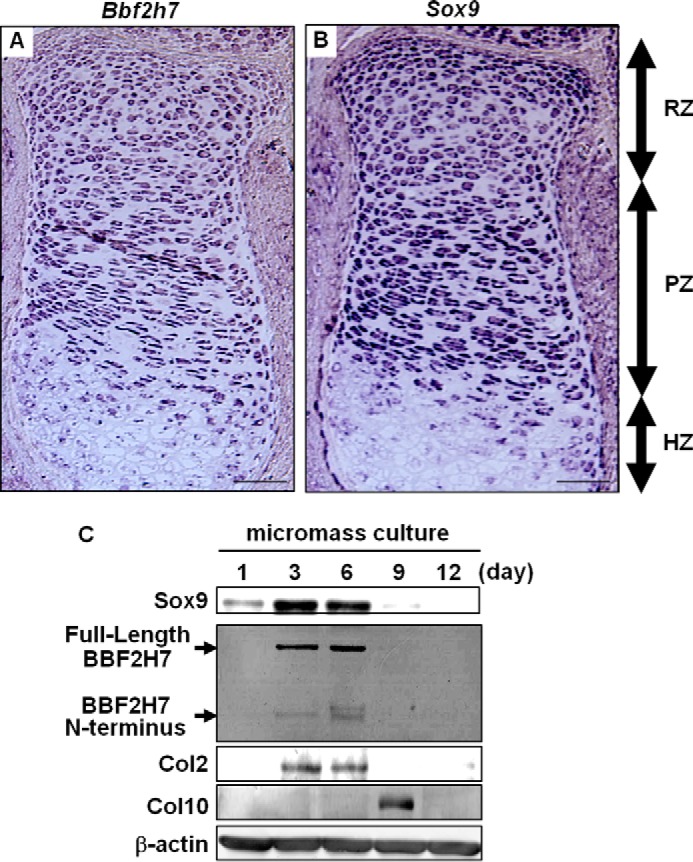
**Both BBF2H7 and Sox9 are expressed in resting chondrocytes and proliferating chondrocytes, but not in hypertrophic chondrocytes.** *A* and *B*, *in situ* hybridization of (*A*) *Bbf2h7* and (*B*) *Sox9* in the serial sections of tibia at E18.5 mice. Both *Bbf2h7* and *Sox9* were expressed in resting chondrocytes and proliferating chondrocytes, but not in hypertrophic chondrocytes in the developing cartilage. *RZ*: resting zone, *PZ*: proliferating zone, *HZ*: hypertrophic zone. Bars: 100 μm. *C*, Western blotting of BBF2H7 in mesenchymal stem cells maintained in micromass culture for the indicated days. Note that the expressions of BBF2H7, Sox9 and its target Col2 were transiently up-regulated on days 3 and 6 with a subsequent reduction. In contrast, the expression of Col10, a hypertrophic chondrocyte marker, was induced on day 9.

BBF2H7 is preferentially expressed in chondrocytes of developing cartilage (15). To examine the expression pattern of *Bbf2h7* in developing cartilage, we performed *in situ* hybridization (Fig. 1*A*). Strong signals of *Bbf2h7* mRNA were detected in resting chondrocytes and proliferating chondrocytes, but not in hypertrophic chondrocytes. These *Bbf2h7* expression patterns were very similar to those of *Sox9*. Indeed, *Sox9* expression was also observed in resting chondrocytes and proliferating chondrocytes, but not in hypertrophic chondrocytes (Fig. 1*B*). Next, we checked the expression of BBF2H7 and Sox9 during chondrocyte differentiation using micromass culture of mesenchymal stem cells prepared from E11.5 WT limbs (Fig. 1*C*). In the micromass cultured cells, the expression of BBF2H7, Sox9 and the target of Sox9, Col2, were relatively low at day 1. These signals markedly increased at day 3 to day 6 with a subsequent reduction. Conversely, the transient up-regulation of Type X collagen (Col10), a hypertrophic chondrocyte marker, was observed on day 9. These results indicate that both BBF2H7 and Sox9 are induced in resting chondrocytes and proliferating chondrocytes, and the expression patterns closely resemble each other during chondrogenesis.

##### Bbf2h7 Is a Target of Sox9 in Chondrocytes

**FIGURE 2.**
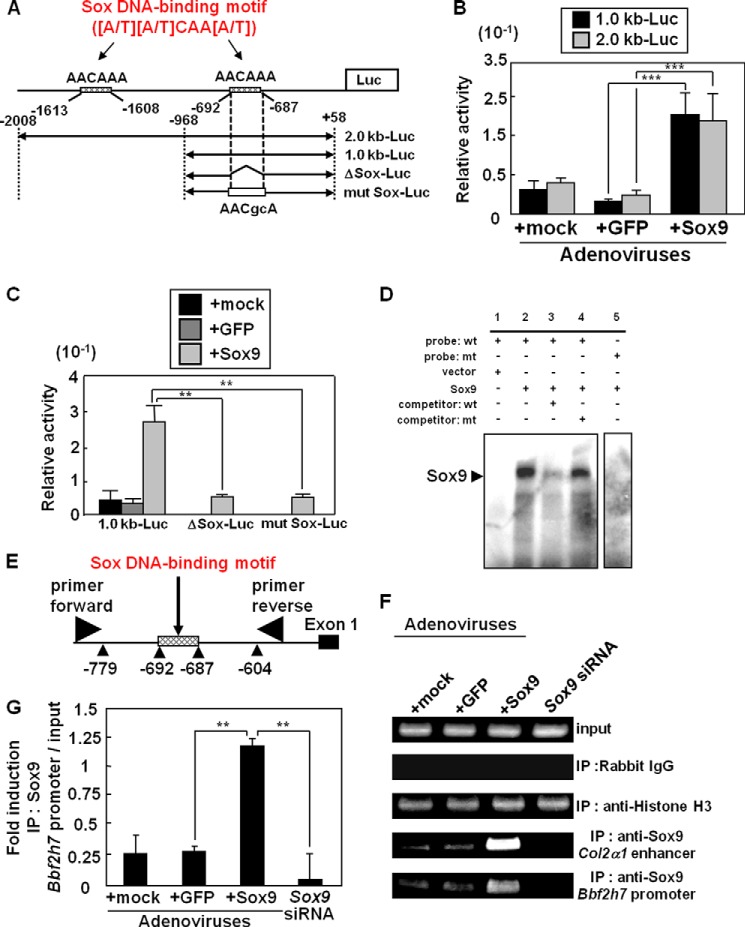
***Bbf2h7* is a direct target of *Sox9* in chondrocytes.** *A*, scheme of the mouse *Bbf2h7* promoter region and reporter constructs. Two Sox DNA-binding motifs ([A/T][A/T]CAA[A/T]) are included within 2.0 kb upstream of the *Bbf2h7* start site (AACAAA: −1613 to −1608 bp and AACAAA: −692 to −687 bp). 2.0 kb-Luc and 1.0 kb-Luc constructs consist of 2.0 kb and 1.0 kb upstream of the *Bbf2h7* start site, respectively. ΔSox-Luc is 1.0 kb-Luc construct lacking the downstream Sox DNA-binding motif (AACAAA: −692 to −687 bp), and mut Sox-Luc is 1.0 kb-Luc construct mutated (small letters in Sox DNA-binding motif) in the downstream Sox DNA-binding motif. Luc: luciferase gene. *B*, reporter assays using ATDC5 cells. Cells transfected with 2.0 kb-Luc or 1.0 kb-Luc constructs in *A* were infected with adenoviruses expressing Sox9 or GFP. GFP was used as a control (*n* = 6). *C*, reporter assays using ATDC5 cells. Cells transfected with 1.0 kb-Luc, ΔSox-Luc, or mut Sox-Luc constructs in *A* were infected with adenoviruses expressing Sox9 or GFP. Note that in ATDC5 cells transfected with ΔSox-Luc or mut Sox-Luc constructs, reporter activities were markedly reduced, despite the expression of Sox9. GFP was used as a control (*n* = 6). *D*, electrophoresis mobility shift assay (EMSA). Note that the binding of Sox9 to the Sox DNA-binding motif was abolished by a competitor (*lane 3*) or mutated probe (*lane 5*). The samples of EMSA were run as part of the same experiment on the same gel. *mt*: mutation. *E*, schematic representation of the *Bbf2h7* promoter and the annealing sites of the primer set used in the chromatin immunoprecipitation assays. *F*, chromatin immunoprecipitation assay using primary cultured chondrocytes infected with adenoviruses expressing Sox9 or GFP. Immunoprecipitation of chromatin was performed using the indicated antibodies. GFP was used as a control. *G*, quantitative analysis of PCR amplification of *Bbf2h7* promoter region after immunoprecipitation by anti-Sox9 antibody in *F* (*n* = 5). Mock indicates empty vector. Values represent mean ± S.D. **, *p* < 0.01; ***, *p* < 0.001.

To investigate the relationship between BBF2H7 and Sox9 expression, we searched for potential binding sites of various transcription factors within 2.0 kb upstream of the *Bbf2h7* and *Sox9* transcription start sites using TFSEARCH. We found two cis-acting elements for Sox9 in the *Bbf2h7* promoter region (AACAAA: −1613 to −1608 bp and AACAAA: −692 to −687 bp) that are conserved among human, mouse and rat. In contrast, the *Sox9* promoter did not contain the conserved cAMP response element (TGACGTTT) that BBF2H7 can bind to (14). To determine whether Sox9 acts on the *Bbf2h7* promoter and activates *Bbf2h7* transcription in chondrocytes, we performed promoter assays using a reporter gene carrying a 2.0 kb (containing two Sox DNA-binding motifs) or 1.0 kb (containing one Sox DNA-binding motif) promoter region of *Bbf2h7* (2.0 kb-Luc and 1.0 kb-Luc, respectively; Fig. 2*A*). In ATDC5 cells (murine chondrogenic cells) transfected with 2.0 kb-Luc or 1.0 kb-Luc constructs, reporter activities were dramatically induced by infection of adenoviruses expressing Sox9 (Fig. 2*B*). These data suggest that Sox9 acts on the downstream Sox DNA-binding motif (-692 to −687 bp) within the *Bbf2h7* promoter. We next performed a promoter assay using the reporter constructs ΔSox-Luc, which lacks the downstream Sox DNA-binding motif, and mut Sox-Luc, which has a mutation in the motif (Fig. 2*A*). In ATDC5 cells transfected with these constructs, reporter activities were markedly reduced, despite the expression of Sox9 (Fig. 2*C*). To confirm that Sox9 directly binds to the downstream Sox DNA-binding motif in the *Bbf2h7* promoter, we performed an electrophoresis mobility shift assay (Fig. 2*D*). We found that Sox9 protein bound to the downstream Sox DNA-binding motif in the *Bbf2h7* promoter. This binding was abolished by incubation with 100-fold excess of unlabeled competitors, but not by mutated competitors. Furthermore, we performed chromatin immunoprecipitation (ChIP) assays using primary cultured chondrocytes infected with adenoviruses expressing Sox9. We detected a high level of Sox9 binding to the Sox DNA-binding motif in the endogenous *Bbf2h7* promoter, as well as to that in the enhancer region of *Col2*α*1*, which is known as a target of Sox9 (32) (Fig. 2, *E–G*). These bindings were abolished in *Sox9*-knockdown chondrocytes, indicating that Sox9 directly acts on Sox DNA-binding motif (−692 to −687 bp) within the *Bbf2h7* promoter and facilitates its transcription in chondrocytes.

##### BBF2H7 Is Up-regulated and Activated as a Transcription Factor by Sox9 Expression

**FIGURE 3.**
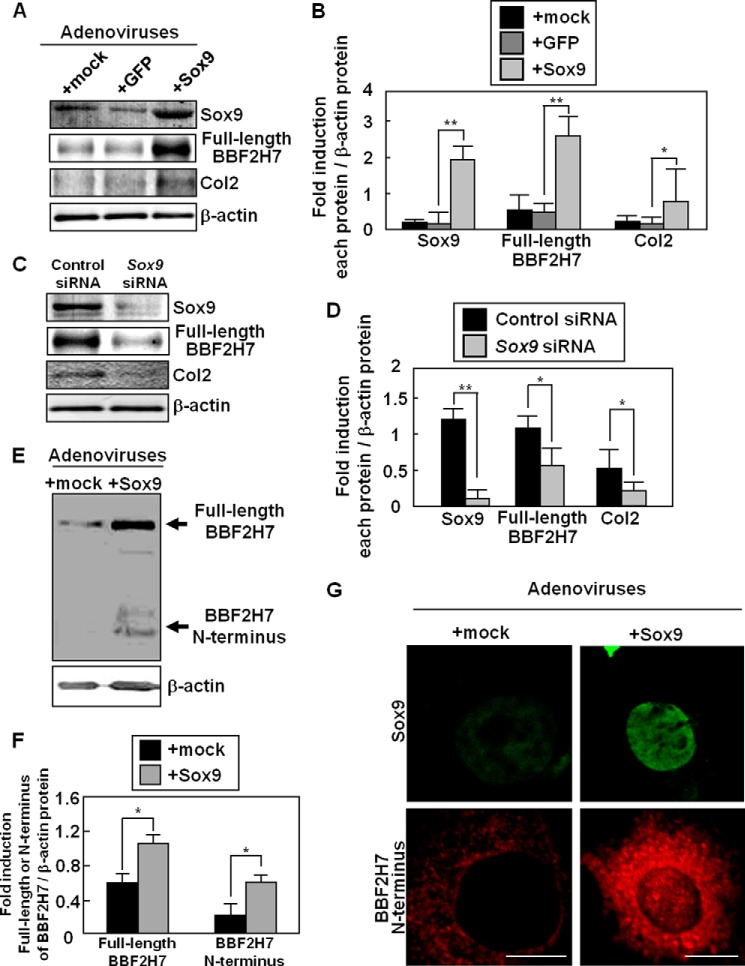
**Up-regulation and activation of BBF2H7 by Sox9.** *A*, western blotting of Sox9, BBF2H7, and Col2 in primary cultured chondrocytes infected with adenoviruses expressing Sox9 or GFP. GFP was used as a control. *B*, quantitative analysis of Sox9, BBF2H7 and Col2 expression in *A*. Data are shown as ratios of each protein and β-actin (*n* = 4). *C*, Western blotting of Sox9, BBF2H7 and Col2 in primary cultured chondrocytes transfected with *Sox9*-targeting siRNA. *D*, quantitative analysis of Sox9, BBF2H7, and Col2 expression in *C*. Data are shown as ratios of each protein and β-actin (*n* = 3). *E*, Western blotting of BBF2H7 in primary cultured chondrocytes infected with adenoviruses expressing Sox9. The amounts of full-length BBF2H7 and the N-terminal fragments were increased by the expression of Sox9. *F*, quantitative analysis of full-length BBF2H7 and the N terminus proteins in *E*. Data are shown as ratios of each protein and β-actin (*n* = 3). *G*, immunostaining of Sox9 (*top panels*, *green*) and BBF2H7 (*bottom panels*, *red*) using primary cultured chondrocytes infected with adenoviruses expressing Sox9. The cleaved BBF2H7 N terminus translocated into the nucleus upon the expression of Sox9. Bars: 5 μm. *Mock* indicates empty vector. Values represent mean ± S.D. *, *p* < 0.05; **, *p* < 0.01.

We compared the expression of BBF2H7 in primary cultured chondrocytes infected with adenoviruses expressing GFP (negative control) or Sox9 (Fig. 3, *A* and *B*). Western blotting analysis showed that the expression of not only Col2 but also BBF2H7 was significantly up-regulated by more than 3-fold. Conversely, BBF2H7 expression was inhibited by transfection with *Sox9*-specific siRNA (Fig. 3, *C* and *D*). These data suggest that the expression of BBF2H7 is regulated by Sox9 in chondrocytes. We next investigated whether BBF2H7 is activated as a transcription factor after the Sox9-induced expression. We have previously reported that abundant synthesis of cartilage matrix proteins causes mild ER stress in the proliferating zone of developing cartilage (15). Infection of primary cultured chondrocytes with adenoviruses expressing Sox9, followed by promotion of synthesis of cartilage matrix proteins, including Col2, caused ER stress (15, 23). BBF2H7 is activated in response to this mild ER stress during chondrogenesis. Furthermore, we have demonstrated that the amount of Full-length BBF2H7 increases by its stabilization under ER stress conditions (21). The large amount of Full-length BBF2H7 is automatically cleaved by S1P and S2P at the Golgi apparatus, and the amount of its N-terminal fragments subsequently increases, followed by translocation into the nucleus. In fact, Western blotting showed that the levels of both full-length and N-terminal fragments of BBF2H7 were increased by expression of Sox9 in primary cultured chondrocytes (Fig. 3, *E* and *F*). In addition, we confirmed the translocation of BBF2H7 N terminus into the nucleus after expression of Sox9 (Fig. 3*G*). Thus, we concluded that BBF2H7 is activated as a transcription factor after its up-regulation by Sox9.

##### Sox9 Promotes the Secretion of Cartilage Matrix Proteins through the Activation of BBF2H7-Sec23a Signaling

**FIGURE 4.**
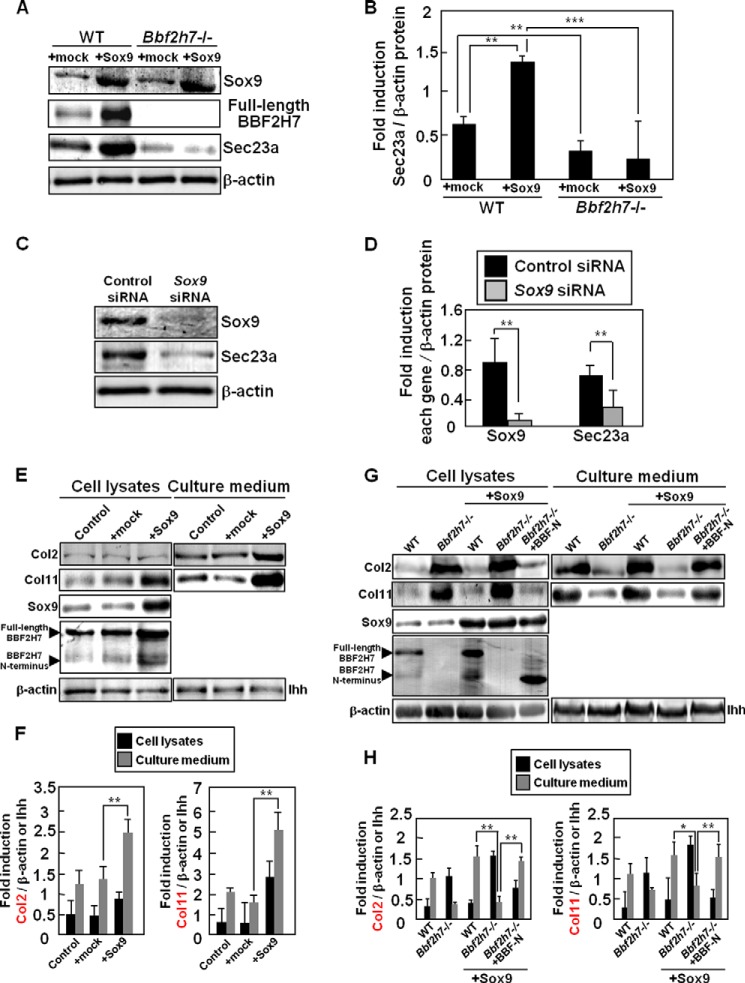
**Sox9 promotes the secretion of cartilage matrix proteins through the activation of BBF2H7-Sec23a signaling.** *A*, Western blotting of Sox9, BBF2H7 and Sec23a in WT and *Bbf2h7*^−/−^ primary cultured chondrocytes infected with adenoviruses expressing Sox9. Note that in *Bbf2h7*^−/−^ primary cultured chondrocytes, the expression of BBF2H7 and Sec23a was inhibited despite the expression of Sox9. *B*, quantitative analysis of Sec23a expression in *A*. Data are shown as ratios of Sec23a and β-actin (*n* = 3). *C*, Western blotting of Sox9 and Sec23a in primary cultured chondrocytes transfected with *Sox9*-targeting siRNA. *D*, quantitative analysis of Sox9 and Sec23a expression in *C*. Data are shown as ratios of each protein and β-actin (*n* = 3). *E*, Western blotting of Col2 and Col11 using cell lysates and culture medium collected from primary cultured chondrocytes infected with adenoviruses expressing Sox9. Note that the amounts of Col2 and Col11 proteins secreted into the extracellular space were increased upon expression of Sox9. Col2, Col11, or Ihh in the culture medium (*right panels*) was immunoprecipitated using anti-Col2, anti-Col11, or anti-Ihh antibodies, respectively. *F*, quantitative analysis of Col2 and Col11 proteins in *E*. Data are shown as ratios of each protein and β-actin (cell lysates) or Ihh (culture medium) (*n* = 4). *G*, Western blotting of Col2 and Col11 using cell lysates and culture medium collected from WT and *Bbf2h7*^−/−^ primary cultured chondrocytes infected with adenoviruses expressing Sox9 and BBF2H7 N terminus. Col2, Col11 or Ihh in the culture medium (*right panels*) was immunoprecipitated using anti-Col2, anti-Col11, or anti-Ihh antibodies, respectively. The secretions of Col2 and Col11 into the extracellular space were inhibited in *Bbf2h7*^−/−^ primary cultured chondrocytes expressing Sox9 alone. *H*, quantitative analysis of Col2 and Col11 proteins in *G*. Data are shown as ratios of each protein and β-actin (cell lysates) or Ihh (culture medium) (*n* = 3). *Mock* indicates empty vector. Values represent mean ± S.D. *, *p* < 0.05; **, *p* < 0.01; ***, *p* < 0.001.

Previously, we have demonstrated that BBF2H7 promotes the expression of Sec23a, which is responsible for protein transport from the ER to the Golgi apparatus and is a target of BBF2H7, followed by acceleration of cartilage matrix protein secretion (15). As shown in Fig. 3, Sox9 induced the expression of BBF2H7, followed by promoting its activation as a transcription factor. Therefore, we next examined whether Sox9 is indirectly involved in the expression of Sec23a through the regulation of BBF2H7 activation (Fig. 4, *A* and *B*). The expression of Sec23a was significantly induced by expression of Sox9 in primary cultured chondrocytes. On the contrary, knockdown of *Sox9* decreased the expression level of Sec23a (Fig. 4, *C* and *D*). Furthermore, the expression was significantly reduced in *Bbf2h7*^−/−^ primary cultured chondrocytes despite the expression of Sox9 (Fig. 4, *A* and *B*), suggesting that Sox9 indirectly up-regulates the expression of Sec23a through the activation of BBF2H7. Our previous report has shown that the secretion of cartilage matrix proteins, including Col2, was inhibited by the deletion of BBF2H7-Sec23a signaling (15). Thus, we examined the amounts of Col2 and Col11, which are both targets of Sox9, in the extracellular space using primary cultured chondrocytes infected with adenoviruses expressing Sox9. The amounts of both total and secreted Col2 and Col11 increased in cells expressing Sox9 (Fig. 4, *E* and *F*). Increased synthesis of these proteins was also observed in *Bbf2h7*^−/−^ cells expressing Sox9 (Fig. 4, *G* and *H*). However, the secretion of Col2 and Col11 was inhibited in this case. Ectopic expression of Sox9 and BBF2H7 N terminus in *Bbf2h7*^−/−^ cells completely rescued the impaired secretion of these proteins. These results suggest that Sox9 directly promotes the synthesis of Col2 and Col11, and indirectly accelerates their secretion into the extracellular space through activation of BBF2H7-Sec23a signaling.

##### Sox9-BBF2H7 Signaling Is Specifically Activated during Chondrogenesis

**FIGURE 5.**
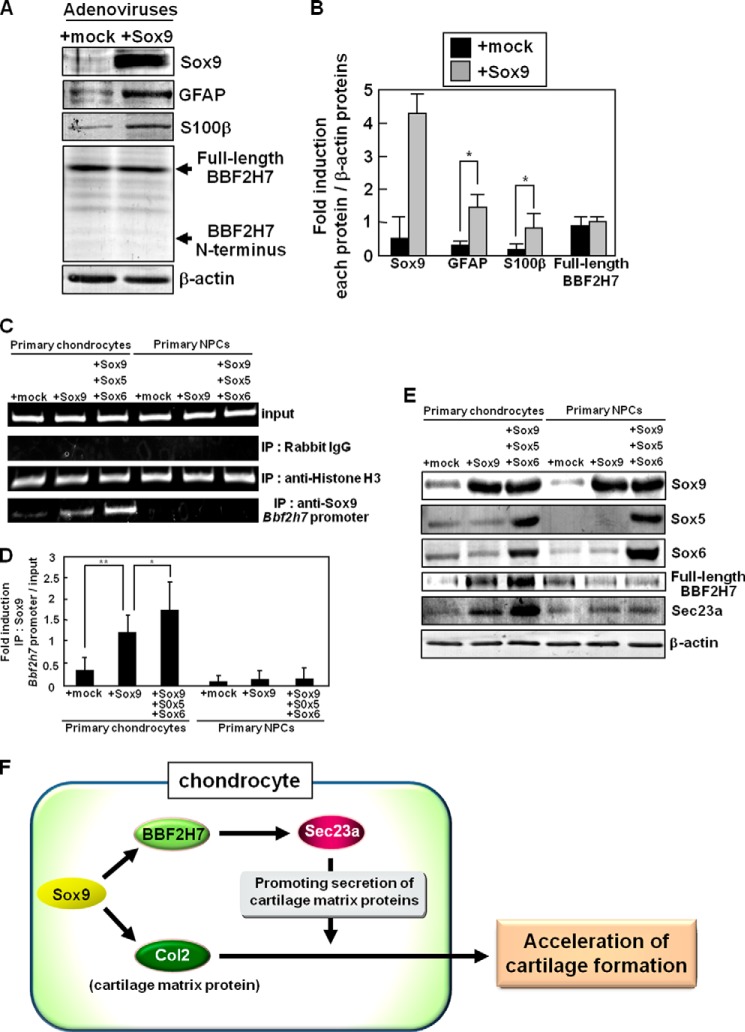
**Sox9-BBF2H7 signaling is not activated during gliogenesis.** *A*, Western blotting of Sox9, GFAP, S100β, and BBF2H7 in primary cultured NPCs infected with adenoviruses expressing Sox9. *B*, quantitative analysis of Sox9, GFAP, S100β, and full-length BBF2H7 proteins in *A*. Data are shown as ratios of each protein and β-actin (*n* = 3). *C*, chromatin immunoprecipitation assay using primary cultured chondrocytes and NPCs infected with adenoviruses expressing Sox9, or Sox5, Sox6, and Sox9. Immunoprecipitation of chromatin was performed using the indicated antibodies. *D*, quantitative analysis of PCR amplification of the *Bbf2h7* promoter region after immunoprecipitation by anti-Sox9 antibody in *C* (*n* = 3). *E*, Western blotting of Sox9, Sox5, Sox6, BBF2H7, and Sec23a using primary cultured chondrocytes and NPCs infected with adenoviruses expressing Sox9, or Sox5, Sox6, and Sox9. Note that the expression of BBF2H7 and Sec23a was significantly induced in primary cultured chondrocytes infected with adenoviruses expressing Sox5, Sox6, and Sox9, but not in primary cultured NPCs. *F*, proposed model for Sox9-BBF2H7-Sec23a pathway to accelerate the secretion of cartilage matrix proteins induced by Sox9. *Mock* indicates empty vector. Values represent mean ± S.D. *, *p* < 0.05; **, *p* < 0.01.

Sox9 plays crucial roles not only in chondrocyte differentiation but also in glial differentiation. The number of glial cells was severely decreased in mice in which Sox9 was specifically ablated from neural precursor cells (NPCs). The expression of Sox9 in Neuro2a cells (murine neuroblastoma) causes concomitant induction of several glial markers (37). It is possible that Sox9-BBF2H7-Sec23a signaling is activated during glial differentiation as well as chondrocyte differentiation. Thus, we examined whether Sox9 promotes the expression of BBF2H7 during glial differentiation using primary cultured NPCs. The protein expressions of glial fibrillary acidic protein (GFAP) and S100β, which are both astrocyte markers, were induced by expressing Sox9 in primary cultured NPCs (Fig. 5, *A* and *B*). However, the expression of BBF2H7 was unchanged in NPCs expressing Sox9. The amount of cleaved BBF2H7 N terminus did not change in these cells. We further examined the regulatory mechanisms of BBF2H7 expression by Sox9 in each distinct cell. It is well known that the formation of a Sox9-transcriptional complex including Sox5 and Sox6, which are also Sox proteins, is essential for promoting the expression of Sox9 targets during chondrogenesis (38). We examined whether Sox5 and Sox6 as well as Sox9 are necessary for inducing the expression of BBF2H7 by performing a ChIP assay and Western blotting analysis using primary cultured chondrocytes and primary cultured NPCs (Fig. 5, *C–E*). In primary cultured chondrocytes expressing Sox5, Sox6, and Sox9, the binding of Sox9 to the *Bbf2h7* promoter was stronger than that in chondrocytes expressing Sox9 only (Fig. 5, *C* and *D*). The expression of BBF2H7 and Sec23a was significantly induced in these cells (Fig. 5*E*). However, these effects were not observed in primary cultured NPCs expressing Sox5, Sox6, and Sox9. These results suggest that Sox5 and Sox6 facilitate the Sox9-regulated expression of BBF2H7 in chondrocytes, but not in NPCs. Taken together, we concluded that the Sox9-transcriptional complex formed by Sox5, Sox6, and Sox9 does not promote the transcriptional activation and cleavage of BBF2H7 during glial differentiation. The transcriptional regulation of BBF2H7 by Sox9-transcriptional complex may be specific to chondrocyte differentiation.

## DISCUSSION

It is well known that Sox9 plays crucial roles in all sequential steps of chondrocyte differentiation (29–31). Recently, we have demonstrated that BBF2H7 is also essential for chondrogenesis (15). Although these two molecules are both significant factors for chondrocyte differentiation and cartilage formation, and show similar expression patterns in developing cartilage, their relationship was not clear. In the present study, we demonstrated that Sox9 acts on the Sox DNA-binding motif within the *Bbf2h7* promoter and facilitates its transcription in chondrocytes. This conclusion is supported by the following findings: 1) BBF2H7 was significantly up-regulated by Sox9 expression and was down-regulated by knockdown of *Sox9* in primary cultured chondrocytes; 2) a Sox DNA-binding motif that Sox9 can bind to exists in the *Bbf2h7* promoter region; 3) overexpression of Sox9 up-regulated *Bbf2h7* promoter activities; 4) *Bbf2h7* promoter activities were drastically decreased by deletion or mutation of the Sox DNA-binding motif; and 5) Sox9 directly binds to Sox DNA-binding motif in the *Bbf2h7* promoter. Sox9 promotes the expressions of Col2 and Col11 as well as BBF2H7, to accelerate chondrocyte differentiation and cartilage formation (32, 33). The syntheses of abundant cartilage matrix proteins, including Col2 and Col11, increase the burden on the ER, leading to BBF2H7 activation (15). Simultaneous induction of these cartilage matrix proteins and BBF2H7 expression by Sox9 may allow the rapid secretion of synthesized Col2 and Col11 during chondrogenesis.

Sox9 acts on the *Bbf2h7* promoter to induce BBF2H7 expression, followed by acceleration of cartilage matrix protein secretion through the activation of BBF2H7-Sec23a signaling during chondrocyte differentiation, but not during glial differentiation. We found that the Sox9-transcriptional complex formed by Sox5, Sox6, and Sox9 promotes the expression of BBF2H7 in chondrocytes. However, this complex did not affect the BBF2H7 expression in NPCs. Although the precise cause of this phenomenon is still unclear, it may be explained by the following possibilities: 1) the expression of *Bbf2h7* may be controlled by epigenetic regulation such as the methylation and acetylation status of the *Bbf2h7* promoter region and histone modifications. We found that Sox9 was hardly able to bind to the Sox DNA-binding motif of the *Bbf2h7* promoter in primary cultured NPCs (Fig. 5*C*). Thus, we speculated that the expression of BBF2H7 by Sox9 may be also regulated by epigenetic regulation. BBF2H7 is expressed in various cells and tissues including chondrocyes, but not ubiquitously (14). Cell- or tissue-specific mechanisms may regulate the epigenetic expression of BBF2H7. 2) Specific co-factors may be necessary for transcriptional activation of *Bbf2h7* by Sox9. It has been reported that Sox9 forms the transcriptional complex including Sox5 and Sox6 to promote the expression of Sox9 targets during chondrogenesis (38). *Sox5* and *Sox6* double-null mutant mice exhibit severe chondrodysplasia characterized by largely arrested development at the stage of chondrogenic mesenchymal condensations and virtual absence of cartilage (39), indicating that Sox5 and Sox6 are also needed for differentiation of chondrocytes. Actually, we found that the expression of BBF2H7 is also controlled by Sox5, Sox6 and Sox9. On the other hand, it is known that Sox9 plays critical roles in the determination of glial fate and controls its differentiation in cooperation with Sox8 and Sox10 instead of Sox5 and Sox6 (32, 37, 40–42, 44). Distinct Sox family proteins may allow Sox9 to induce the expression of diverse target genes in each cell. Further studies are necessary to elucidate the molecular mechanisms for selective transcriptional activation of *Bbf2h7* in each cell and tissue type.

Ihh, which is expressed in prehypertrophic chondrocytes of developing cartilage, diffuses to the periarticular chondrocytes to induce the expression of PTHrP (45–47). The Ihh-PTHrP pathway promotes the proliferation of proliferating chondrocytes and inhibits hypertrophic differentiation. It has been reported that deletion of *Sox9* in chondrocytes at later stages of development resulted in decreased proliferation of proliferating chondrocytes and increased numbers of premature hypertrophic chondrocytes (30). These findings suggest that the signaling downstream of Ihh may be impaired by *Sox9* deficiency. Indeed, the expression of *Patched-1 (Ptch1*), a Hedgehog (Hh) receptor and the transcriptional target of Ihh signaling, was inhibited in the developing cartilage of *Sox9*-deletion mice. The expression of PTHrP was also clearly down-regulated in these mice (30). Previous reports have shown that Sox5, Sox6, and Sox9 bound to the promoter region of *Pthrp* and up-regulated its promoter activity (43). However, the detailed cause of these abnormalities is not well understood. In a recent study, we have found similar phenotypes in the developing cartilage of *Bbf2h7*^−/−^ mice (23). We have demonstrated that the cleaved BBF2H7 C-terminal fragments are secreted from proliferating chondrocytes into the extracellular space to promote the proliferation of neighboring cells to act as a co-factor for the Ihh-Ptch1 complex, followed by activating Hh signaling (23). The expressions of Hh targets, including Ptch1, were suppressed in *Bbf2h7*^−/−^ mice. It is possible that disruption of the Ihh-PTHrP pathway followed by inhibiting the proliferation of proliferating chondrocytes and increase in the number of premature hypertrophic chondrocytes by the absence of *Sox9* may be partially involved in the reduction in BBF2H7 expression. Further studies are required to clarify whether Sox9-BBF2H7 signaling could contribute to the growth of developing cartilage through regulation of Ihh signaling as well as the acceleration of cartilage matrix protein secretion.

In conclusion, Sox9 simultaneously promotes transcriptional activation of *Bbf2h7* and cartilage matrix genes, including *Col2*α*1*. BBF2H7 is activated in response to physiological ER stress caused by the abundant synthesis of cartilage matrix proteins (15), followed by promoting the secretion of these proteins through induction of Sec23a expression (Fig. 5*F*). The present study demonstrated novel regulatory mechanisms of the secretory pathway downstream of Sox9. Our findings expand the significant roles of Sox9 from a master regulator for promoting the expression of cartilage matrix genes, to a crucial factor for regulating the efficient secretion of these synthesized proteins through direct activation of the BBF2H7-Sec23a pathway.

## Footnotes

3 The abbreviations used are:

ER: endoplasmic reticulum
SRY: Sex determining region Y
HMG: high-mobility group
Sox9: Sex determining region Y-related high-mobility group box 9
BBF2H7: box B-binding factor-2 human homolog on chromosome 7
bZIP: basic leucine zipper
IRE1: inositol-requiring kinase 1
ATF6: activating transcription factor 6
UPR: unfolded protein response
OASIS: old astrocyte specifically induced substance
CREB: cyclic AMP response element-binding protein
E: embryonic day
S1P: site-1-protease
Col: collagen
Hh: Hedgehog
Ihh: Indian hedgehog
PTHrP: parathyroid hormone-related protein
NPCs: neural precursor cells
GFAP: glial fibrillary acidic protein
Sox: sex-determining region Y-related high-mobility
DEPC: diethylpyrocarbonate
SSC: saline sodium citrate.

## References

[1] Ron D. (2002) Translational control in the endoplasmic reticulum stress response. J. Clin. Invest. 110, 1383–1388 12438433 10.1172/JCI16784 PMC151821

[2] Kaufman R. J. (2002) Orchestrating the unfolded protein response in health and disease. J. Clin. Invest. 110, 1389–1398 12438434 10.1172/JCI16886 PMC151822

[3] Reimold A. M. Iwakoshi N. N. Manis J. Vallabhajosyula P. Szomolanyi-Tsuda E. Gravallese E. M. Friend D. Grusby M. J. Alt F. Glimcher L. H. (2001) Plasma cell differentiation requires the transcription factor XBP-1. Nature 412, 300–307 11460154 10.1038/35085509

[4] Zhang K. Shen X. Wu J. Sakaki K. Saunders T. Rutkowski D. T. Back S. H. Kaufman R. J. (2006) Endoplasmic reticulum stress activates cleavage of CREBH to induce a systemic inflammatory response. Cell 124, 587–599 16469704 10.1016/j.cell.2005.11.040

[5] Vecchi C. Montosi G. Zhang K. Lamberti I. Duncan S. A. Kaufman R. J. Pietrangelo A. (2009) ER stress controls iron metabolism through induction of hepcidin. Science 325, 877–880 19679815 10.1126/science.1176639 PMC2923557

[6] Harding H. P. Zhang Y. Ron D. (1999) Protein translation and folding are coupled by an endoplasmic-reticulum-resident kinase. Nature 397, 271–274 9930704 10.1038/16729

[7] Tirasophon W. Welihinda A. A. Kaufman R. J. (1998) A stress response pathway from the endoplasmic reticulum to the nucleus requires a novel bifunctional protein kinase/endoribonuclease (Ire1p) in mammalian cells. Genes Dev. 12, 1812–1824 9637683 10.1101/gad.12.12.1812 PMC316900

[8] Calfon M. Zeng H. Urano F. Till J. H. Hubbard S. R. Harding H. P. Clark S. G. Ron D. (2002) IRE1 couples endoplasmic reticulum load to secretory capacity by processing the XBP-1 mRNA. Nature 415, 92–96 11780124 10.1038/415092a

[9] Yoshida H. Okada T. Haze K. Yanagi H. Yura T. Negishi M. Mori K. (2000) ATF6 activated by proteolysis binds in the presence of NF-Y (CBF) directly to the cis-acting element responsible for the mammalian unfolded protein response. Mol. Cell. Biol. 20, 6755–6767 10958673 10.1128/mcb.20.18.6755-6767.2000 PMC86199

[10] Asada R. Kanemoto S. Kondo S. Saito A. Imaizumi K. (2011) The signaling from endoplasmic reticulum-resident bZIP transcription factors involved in diverse cellular physiology. J. Biochem. 149, 507–518 21454302 10.1093/jb/mvr041

[11] Kondo S. Saito A. Asada R. Kanemoto S. Imaizumi K. (2011) Physiological unfolded protein response regulated by CREB/ATF family members, transmembrane bZIP transcription factors. IUBMB Life 63, 233–239 21438114 10.1002/iub.433

[12] Kondo S. Murakami T. Tatsumi K. Ogata M. Kanemoto S. Otori K. Iseki K. Wanaka A. Imaizumi K. (2005) OASIS, a CREB/ATF-family member, modulates UPR signalling in astrocytes. Nat. Cell Biol. 7, 186–194 15665855 10.1038/ncb1213

[13] Murakami T. Saito A. Hino S-I. Kondo S. Kanemoto S. Chihara K. Sekiya H. Tsumagari K. Ochiai K. Yoshinaga K. Saitoh M. Nishimura R. Yoneda T. Kou I. Furuichi T. Ikegawa S. Ikawa M. Okabe M. Wanaka A. Imaizumi K. (2009) Signalling mediated by the endoplasmic reticulum stress transducer OASIS is involved in bone formation. Nat. Cell Biol. 11, 1205–1211 19767743 10.1038/ncb1963

[14] Kondo S. Saito A. Hino S-I. Murakami T. Ogata M. Kanemoto S. Nara S. Yamashita A. Yoshinaga K. Hara H. Imaizumi K. (2007) BBF2H7, a novel transmembrane bZIP transcription factor, is a new type of endoplasmic reticulum stress transducer. Mol. Cell. Biol. 27, 1716–1729 17178827 10.1128/MCB.01552-06 PMC1820470

[15] Saito A. Hino S-I. Murakami T. Kanemoto S. Kondo S. Saitoh M. Nishimura R. Yoneda T. Furuichi T. Ikegawa S. Ikawa M. Okabe M. Imaizumi K. (2009) Regulation of endoplasmic reticulum stress response by a BBF2H7-mediated Sec23a pathway is essential for chondrogenesis. Nat. Cell Biol. 11, 1197–1204 19767744 10.1038/ncb1962

[16] Omori Y. Imai J. Watanabe M. Komatsu T. Suzuki Y. Kataoka K. Watanabe S. Tanigami A. Sugano S. (2001) CREB-H: a novel mammalian transcription factor belonging to the CREB/ATF family and functioning via the box-B element with a liver-specific expression. Nucleic Acids Res. 29, 2154–2162 11353085 10.1093/nar/29.10.2154 PMC55463

[17] Cao G. Ni X. Jiang M. Ma Y. Cheng H. Guo L. Ji C. Gu S. Xie Y. Mao Y. (2002) Molecular cloning and characterization of a novel human cAMP response element-binding (CREB) gene (CREB4). J. Hum. Genet. 47, 373–376 12111373 10.1007/s100380200053

[18] Adham I. M. Eck T. J. Mierau K. Müller N. Sallam M. A. Paprotta I. Schubert S. Hoyer-Fender S. Engel W. (2005) Reduction of spermatogenesis but not fertility in Creb3l4-deficient mice. Mol. Cell. Biol. 25, 7657–7664 16107712 10.1128/MCB.25.17.7657-7664.2005 PMC1190296

[19] Lu R. Yang P. O'Hare P. Misra V. (1997) Luman, a new member of the CREB/ATF family, binds to herpes simplex virus VP16-associated host cellular factor. Mol. Cell. Biol. 17, 5117–5126 9271389 10.1128/mcb.17.9.5117 PMC232362

[20] Storlazzi C. T. Mertens F. Nascimento A. Isaksson M. Wejde J. Brosjo O. Mandahl N. Panagopoulos I. (2003) Fusion of the *FUS* and *BBF2H7* genes in low grade fibromyxoid sarcoma. Hum. Mol. Genet. 12, 2349–2358 12915480 10.1093/hmg/ddg237

[21] Kondo S. Hino S-I. Saito A. Kanemoto S. Kawasaki N. Asada R. Izumi S. Iwamoto H. Oki M Miyagi H. Kaneko M. Nomura Y. Urano F. Imaizumi K. (2012) Activation of OASIS family, ER stress transducers, is dependent on its stabilization. Cell Death Differ. 19, 1939–1949 22705851 10.1038/cdd.2012.77 PMC3504707

[22] Lui W. O. Zeng L. Rehrmann V. Deshpande S. Tretiakova M. Kaplan E. L. Leibiger I. Leibiger B. Enberg U. Höög A. Larsson C. Kroll T. G. (2008) CREB3L2-PPARγ fusion mutation identifies a thyroid signaling pathway regulated by intramembrane proteolysis. Cancer Res. 68, 7156–7164 18757431 10.1158/0008-5472.CAN-08-1085

[23] Saito A. Kanemoto S. Zhang Y. Asada R. Hino K. Imaizumi K. (2014) Chondrocyte Proliferation Regulated by Secreted Luminal Domain of ER Stress Transducer BBF2H7/CREB3L2. Mol. Cell 53, 127–139 24332809 10.1016/j.molcel.2013.11.008

[24] Van de Wetering M. Oosterwegel M. van Norren K. Clevers H. (1993) Sox-4, an Sry-like HMG box protein, is a transcription activator in lymphocytes. EMBO J. 12, 3847–3854 8404853 10.1002/j.1460-2075.1993.tb06063.x PMC413668

[25] Hosking B. M. Muscat G. E. O. Koopman P. A. Dowhan D. H. Dunn T. L. (1995) *Trans*-activation and DNA-binding properties of the transcription factor, Sox-18. Nucleic Acids Res. 23, 2626–2628 7651823 10.1093/nar/23.14.2626 PMC307084

[26] Tchénio T. Casella J. F. Heidmann T. (2000) Members of the SRY family regulate the human LINE retrotransposons. Nucleic Acids Res. 28, 411–415 10606637 10.1093/nar/28.2.411 PMC102531

[27] Ng L. J. Wheatley S. Muscat G. E. Conway-Campbell J. Bowles J. E. Bell D. M. Tam P. P. Cheah K. S. Koopman P. (1997) SOX9 binds DNA, activates transcription, and coexpresses with type II collagen during chondrogenesis in the mouse. Dev. Biol. 183, 108–121 9119111 10.1006/dbio.1996.8487

[28] Zhao Q. Eberspaecher H. Lefebvre V. De Crombrugghe B. (1997) Parallel expression of Sox9 and Col2a1 in cells undergoing chondrogenesis. Dev. Dyn. 209, 377–386 9264261 10.1002/(SICI)1097-0177(199708)209:4<377::AID-AJA5>3.0.CO;2-F

[29] Bi W. Deng J. M. Zhang Z. Behringer R. R. de Crombrugghe B. (1999) Sox9 is required for cartilage formation. Nat. Genet. 22, 85–89 10319868 10.1038/8792

[30] Akiyama H. Chaboissier M. C. Martin J. F. Schedl A. de Crombrugghe B. (2002) The transcription factor Sox9 has essential roles in successive steps of the chondrocyte differentiation pathway and is required for expression of *Sox5* and *Sox6*. Genes Dev. 16, 2813–2828 12414734 10.1101/gad.1017802 PMC187468

[31] Kronenberg H. M. (2003) Developmental regulation of the growth plate. Nature 423, 332–336 12748651 10.1038/nature01657

[32] Lefebvre V. Huang W. Harley V. R. Goodfellow P. N. de Crombrugghe B. (1997) SOX9 is a potent activator of the chondrocyte-specific enhancer of the pro α1(II) collagen gene. Mol. Cell. Biol. 17, 2336–2346 9121483 10.1128/mcb.17.4.2336 PMC232082

[33] Bridgewater L. C. Lefebvre V. de Crombrugghe B. (1998) Chondrocyte-specific enhancer elements in the Col11a2 gene resemble the Col2a1 tissue-specific enhancer. J. Biol. Chem. 273, 14998–15006 9614107 10.1074/jbc.273.24.14998

[34] Xie W. F. Zhang X. Sakano S. Lefebvre V. Sandell L. J. (1999) Trans-activation of the mouse cartilage-derived retinoic acid-sensitive protein gene by Sox9. J. Bone Miner. Res. 14, 757–763 10320524 10.1359/jbmr.1999.14.5.757

[35] Sekiya I Tsuji K Koopman P Watanabe H Yamada Y Shinomiya K Nifuji A Noda M (2000) SOX9 enhances aggrecan gene promoter/enhancer activity and is up-regulated by retinoic acid in a cartilage-derived cell line, TC6. J. Biol. Chem. 275, 10738–10744 10753864 10.1074/jbc.275.15.10738

[36] Saito A. Kanemoto S. Kawasaki N. Asada R. Iwamoto H. Oki M. Miyagi H. Izumi S. Sanosaka T. Nakashima K. Imaizumi K. (2012) Unfolded protein response, activated by OASIS family transcription factors, promotes astrocyte differentiation. Nat. Commun. 3, 967 22828627 10.1038/ncomms1971

[37] Stolt C. C. Lommes P. Sock E. Chaboissier M. C. Schedl A. Wegner M. (2003) The Sox9 transcription factor determines glial fate choice in the developing spinal cord. Genes Dev. 17, 1677–1689 12842915 10.1101/gad.259003 PMC196138

[38] Lefebvre V. Li P. de Crombrugghe B. (1998) A new long form of Sox5 (L-Sox5) Sox6 and Sox9 are coexpressed in chondrogenesis and cooperatively activate the type II collagen gene. EMBO J. 17, 5718–5733 9755172 10.1093/emboj/17.19.5718 PMC1170900

[39] Smits P. Li P. Mandel J. Zhang Z. Deng J. M. Behringer R. R. de Crombrugghe B. Lefebvre V. (2001) The transcription factors L-Sox5 and Sox6 are essential for cartilage formation. Dev. Cell 1, 277–290 11702786 10.1016/s1534-5807(01)00003-x

[40] Herbarth B. Pingault V. Bondurand N. Kuhlbrodt K. Hermans-Borgmeyer I. Puliti A. Lemort N. Goossens M. Wegner M. (1998) Mutation of the Sry-related Sox10 gene in Dominant megacolon, a mouse model for human Hirschsprung disease. Proc. Natl. Acad. Sci. U.S.A. 95, 5161–5165 9560246 10.1073/pnas.95.9.5161 PMC20231

[41] Southard-Smith E. M. Kos L. Pavan W. J. (1998) Sox10 mutation disrupts neural crest development in Dom Hirschsprung mouse model. Nat. Genet. 18, 60–64 9425902 10.1038/ng0198-60

[42] Britsch S. Goerich D. E. Riethmacher D. Peirano R. I. Rossner M. Nave K. A. Birchmeier C. Wegner M. (2001) The transcription factor Sox10 is a key regulator of peripheral glial development. Genes Dev. 15, 66–78 11156606 10.1101/gad.186601 PMC312607

[43] Amano K. Hata K. Sugita A. Takigawa Y. Ono K. Wakabayashi M. Kogo M. Nishimura R. Yoneda T. (2009) Sox9 family members negatively regulate maturation and calcification of chondrocytes through up-regulation of parathyroid hormone-related protein. Mol. Biol. Cell 20, 4541–4551 19759178 10.1091/mbc.E09-03-0227 PMC2770942

[44] Stolt C. C. Rehberg S. Ader M. Lommes P. Riethmacher D. Schachner M. Bartsch U. Wegner M. (2002) Terminal differentiation of myelin-forming oligodendrocytes depends on the transcription factor Sox10. Genes Dev. 16, 165–170 11799060 10.1101/gad.215802 PMC155320

[45] Vortkamp A. Lee K. Lanske B. Segre G. V. Kronenberg H. M. Tabin C. J. (1996) Regulation of rate of cartilage differentiation by Indian hedgehog and PTH-related protein. Science 273, 613–622 8662546 10.1126/science.273.5275.613

[46] Lanske B. Karaplis A. C. Lee K. Luz A. Vortkamp A. Pirro A. Karperien M. Defize L. H. Ho C. Mulligan R. C. Abou-Samra A. B. Jüppner H. Segre G. V. Kronenberg H. M. (1996) PTH/PTHrP receptor in early development and Indian hedgehog-regulated bone growth. Science 273, 663–666 8662561 10.1126/science.273.5275.663

[47] St-Jacques B. Hammerschmidt M. McMahon A. P. (1999) Indian hedgehog signaling regulates proliferation and differentiation of chondrocytes and is essential for bone formation. Genes Dev. 13, 2072–2086 10465785 10.1101/gad.13.16.2072 PMC316949

